# Neural and Genetic Correlates of the Social Sharing of Happiness

**DOI:** 10.3389/fnins.2017.00718

**Published:** 2017-12-19

**Authors:** Masahiro Matsunaga, Hiroaki Kawamichi, Tomohiro Umemura, Reiko Hori, Eiji Shibata, Fumio Kobayashi, Kohta Suzuki, Keiko Ishii, Yohsuke Ohtsubo, Yasuki Noguchi, Misaki Ochi, Hidenori Yamasue, Hideki Ohira

**Affiliations:** ^1^Department of Health and Psychosocial Medicine, Aichi Medical University School of Medicine, Nagakute, Japan; ^2^Division of Cerebral Integration, Department of System Neuroscience, National Institute for Physiological Sciences, Okazaki, Japan; ^3^Department of Psychology, Graduate School of Humanities, Kobe University, Kobe, Japan; ^4^Department of Psychiatry, Hamamatsu University School of Medicine, Hamamatsu, Japan; ^5^Department of Cognitive and Psychological Sciences, Graduate School of Informatics, Nagoya University, Nagoya, Japan

**Keywords:** happiness, mentalizing/theory-of-mind network, serotonin 2A receptor gene polymorphism, functional magnetic resonance imaging, vignette-based questionnaire

## Abstract

Happiness is regarded as one of the most fundamental human goals. Given recent reports that positive feelings are contagious (e.g., the presence of a happy person enhances others' happiness) because of the human ability to empathize (i.e., sharing emotions), empathic ability may be a key factor in increasing one's own subjective level of happiness. Based on previous studies indicating that a single nucleotide polymorphism in the serotonin 2A receptor gene [*HTR2A* rs6311 guanine (G) vs. adenine (A)] is associated with sensitivity to emotional stimuli and several mental disorders such as depression, we predicted that the polymorphism might be associated with the effect of sharing happiness. To elucidate the neural and genetic correlates of the effect of sharing happiness, we first performed functional magnetic resonance imaging (fMRI) during a “happy feelings” evocation task (emotional event imagination task), during which we manipulated the valence of the imagined event (positive, neutral, or negative), as well as the presence of a friend experiencing a positive-valence event (presence or absence). We recruited young adult women for this fMRI study because empathic ability may be higher in women than in men. Participants felt happier (*p* < 0.01) and the mentalizing/theory-of-mind network, which spans the medial prefrontal cortex, temporoparietal junction, temporal poles, and precuneus, was significantly more active (*p* < 0.05) in the presence condition than in the absence condition regardless of event valence. Moreover, participants with the GG (*p* < 0.01) and AG (*p* < 0.05) genotypes of *HTR2A* experienced happier feelings as well as greater activation of a part of the mentalizing/theory-of-mind network (*p* < 0.05) during empathy for happiness (neutral/presence condition) than those with the AA genotype. In a follow-up study with a vignette-based questionnaire conducted in a relatively large sample, male and female participants were presented with the same imagined events wherein their valence and the presence of a friend were manipulated. Results showed genetic differences in happiness-related empathy regardless of sex (*p* < 0.05). Findings suggest that *HTR2A* polymorphisms are associated with the effect of sharing happiness by modulating the activity of the mentalizing/theory-of-mind network.

## Introduction

From the time of Aristotle to the modern age, humans have continued to explore what makes us happy, as happiness has always been regarded as one of the most fundamental human goals. Happiness has usually been conceptualized using one of two classifications. Happiness as a trait comprises relatively stable individual differences in levels of happiness, while happiness as a state comprises ephemeral positive feelings or a lack of negative feelings (Schimmack, 2008; Berridge and Kringelbach, 2011). Although the level of happiness is relatively stable within individuals (i.e., trait-like), it also changes within a relatively short period in response to various external factors. For example, studies have indicated that repetitive experiences of hedonic events, which evoke positive feelings, elevate subjective levels of happiness (Seligman et al., 2005; Otake et al., 2006; Schimmack, 2008). In addition, personal relationships have long been considered to be one of the most important modulators of subjective happiness (Saphire-Bernstein and Taylor, 2013), as we experience many hedonic events through personal relationships. These events include praise or kindness received from others (Otake et al., 2006; Kawamichi et al., 2013a).

Interestingly, recent studies have suggested that levels of subjective happiness can be influenced not only by personal positive feelings, but also by the positive feelings of others. Previous studies have indicated that our own emotional states are strongly influenced by the presence of companions (Hatfield et al., 1993; Doherty et al., 1995; Wagner et al., 2015). For example, a recent epidemiological study reported that happiness could spread from person to person. That is, individuals who are surrounded by happy people are more likely to experience future increases in their levels of subjective happiness (Fowler and Christakis, 2008). Another epidemiological study indicated that one's positive feelings could be transferred to others via the social networking website Facebook (Kramer et al., 2014). Such interpersonal emotional processes are termed the “social sharing of emotions” (Rimé et al., 1991). Positive modulation of subjective happiness by the happiness of others may be explained by the human ability to empathize, which allows us to share and understand each other's internal states, beliefs, and intentions (Morelli et al., 2014, 2015; Mascaro et al., 2015). Therefore, empathic ability may be one of the key factors for increasing one's own subjective level of happiness.

Researchers in the field of social neuroscience have identified several distinct brain circuits engaged in inferring and evaluating others' mental states. These circuits include the mentalizing/theory-of-mind system, mirror neuron system, and motion detection system (Cross et al., 2016; Isoda, 2016). Morelli and colleagues have reported that the mentalizing/theory-of-mind system is strongly activated when compared to other empathy-related brain circuits, such as the mirror neuron system, when individuals experience empathy for happiness (Morelli et al., 2014, 2015). The mentalizing/theory-of-mind system, which spans the medial prefrontal cortex (mPFC), temporoparietal junction (TPJ), temporal poles, and precuneus, plays an important role in the prediction of others' intentions. This is because the mPFC acts as an event simulator (elator) that encompasses a multi-modal representation of social event knowledge distributed throughout associated modality-specific areas (Gallagher et al., 2000; Northoff and Bermpohl, 2004; Platek et al., 2004; Buckner and Carroll, 2007; Krueger et al., 2009; Nakao et al., 2012; Roy et al., 2012). Recent structural and functional magnetic resonance imaging (MRI) studies have also reported an association between subjective happiness and the mentalizing/theory-of-mind system (Sato et al., 2015; Matsunaga et al., 2016). The association between happiness and the mentalizing/theory-of-mind system is conceivable because the mPFC plays a pivotal role in future reward estimation (Tanaka et al., 2004; Rushworth et al., 2011), which may be a key factor in enhancing subjective happiness (Sheldon and Houser-Marko, 2001; Lyubomirsky et al., 2005). Because understanding others' happiness likely requires the integration of complex contextual information, including future reward estimation, activity in the mentalizing/theory-of-mind network may be strongly associated with empathy for the happiness of others.

Another study implicates the involvement of the serotonin (5-hydroxytryptamine, 5-HT) system in empathic ability. Researchers have reported that the single nucleotide polymorphism (SNP) adenine (A)-1438 guanine (G) (rs6311) in the serotonin 2A receptor gene (*HTR2A*) is associated with depressive symptoms and increased reactivity to emotional stimuli (Lee and Ham, 2008; Lebe et al., 2013). The amygdala in individuals with the AA genotype is activated more intensely in response to sad facial expressions than it is in G allele carriers (Lee and Ham, 2008). In agreement with this finding, a previous study indicated that patients with depression have higher frequencies of the A allele of A-1438G than the general population (Lebe et al., 2013). Recent studies have also reported an association between impulsivity, which has been proposed as a risk factor for depressive symptoms, and the *HTR2A* polymorphism A-1438G (Nomura et al., 2006; Ishii et al., 2018). Fisher et al. (2009) have reported that serotonin 2A receptor density within the mPFC is associated with negative stimulus-related amygdala activity, suggesting that neural mechanisms including the mPFC underlie the association between the *HTR2A* polymorphism A-1438G and mental disorders of mood and affect. Although these studies may seem to indicate that the serotonin system is associated with susceptibility to negative events/emotions, it appears to be related to empathy in general, including empathy with others' positive feelings. Some studies have indicated that the *HTR2A* polymorphism A-1438G is associated with autism spectrum disorders, which are believed to be associated with empathic ability (Hranilovic et al., 2010; Smith et al., 2014). Moreover, an interesting recent study reported that administration of lysergic acid diethylamide, which is a serotonin 2A receptor agonist, to healthy participants not only produced feelings of happiness, trust, and closeness to others, but also increased emotional empathic ability, as assessed using the Multifaceted Empathy Test (Dolder et al., 2016). We have also previously reported that salivary serotonin levels are associated with empathic abilities and the effect of sharing happiness (Matsunaga et al., 2017). Based on these observations, we anticipated that the functional *HTR2A* polymorphism A-1438G is associated with individual differences in happiness-related empathy, and that *HTR2A* G allele carriers may have high susceptibility to others' happiness. Kimura et al. (2007) have reported that sensitivity to others' happiness varies in individuals and that people who tend to be susceptible to others' happiness exhibit fewer depressive symptoms. Accordingly, we anticipate that the presence of happiness in others leads to an ephemeral increase in happiness levels in individuals who have high empathic ability (or are susceptible to others' happiness).

Considering the above findings, we predicted that *HTR2A* polymorphisms are associated with the effect of sharing happiness via the modulation of the activities of the mentalizing/theory-of-mind network. In order to test this prediction, we conducted a functional magnetic resonance imaging (fMRI) experiment in order to determine blood-oxygen-level-dependent (BOLD) responses during a happy-feelings evocation task. To eliminate the possible confounding effects of sex, age, and psychiatric disease (Doherty et al., 1995; Hranilovic et al., 2010; Beadle et al., 2012; Smith et al., 2014), we focused on healthy female university students in this study. Although women tend to have higher empathic ability than men (Doherty et al., 1995), they exhibit reasonable levels of variance in empathy. Therefore, the analyses were unlikely to be affected by the range restriction problem. However, we admit that generalizability of the results is limited due to the female-only sample. Thus, in order to examine whether the association between *HTR2A* and the effect of sharing happiness is moderated by sex, we subsequently conducted a vignette-based questionnaire experiment involving both men and women. This questionnaire experiment was also used to test the replicability of the study in a relatively large sample. A previous study reported an association between body mass index (BMI) and serotonin 2A receptor expression in the human brain (Erritzoe et al., 2009). Erritzoe and their colleagues reported that BMI is positively correlated with cerebral cortex serotonin 2A receptor binding using positron emission tomography (PET) and the radioactive serotonin 2A receptor ligand ^18^F-altanserin. This suggests that increased food intake and body weight are associated with increased cerebral 5-HT2A receptor expression. Thus, we also considered BMI as a possible confounding factor in the association between *HTR2A* polymorphism and happiness-related empathy. Furthermore, we compared the participants' subjective happiness levels according to *HTR2A* genotype using the questionnaire, as previous findings suggest that enhancing an individual's sensitivity to the happiness of others may increase that individual's own subjective level of happiness (Kimura et al., 2007).

## Materials and methods

### fMRI experiment

#### Participants

We recruited 29 pairs of female friends (58 healthy female volunteers, mean age: 20.8 years, age range: 18–28 years, all exclusively right-handed) following the study's approval by the Ethics Committee of Aichi Medical University (approval number: 14-036). All participants provided written informed consent in accordance with the Declaration of Helsinki. Fifty-seven of the participants were Japanese undergraduate and graduate students in the Tokai area of Japan, while one participant was a working adult. This sample size was determined based on the results of a previous fMRI study (Lee and Ham, 2008). The mean BMI of the participants was 20.1 kg/m^2^ (range: 15.2–27.0). The participants thus had a normal weight range. Because no participants smoked and only five participants consumed alcohol, we discounted these confounding factors, as well as age and BMI, in subsequent analyses. Four participants did not enter the MRI gantry for the following reasons: metallic materials, such as bone fixation bolts, nail art, or permanent make-up were worn (*n* = 3), or nausea was experienced (*n* = 1). Additionally, the *HTR2A* genotype of one participant was not determined. Thus, we excluded five participants from the analysis. Furthermore, three participants exhibited excessive body movements (over 3 mm) during the fMRI experiment. Therefore, we analyzed behavioral data from 53 participants and fMRI data from 50 participants.

#### Experimental task and procedure for fMRI

**Figure 1:**
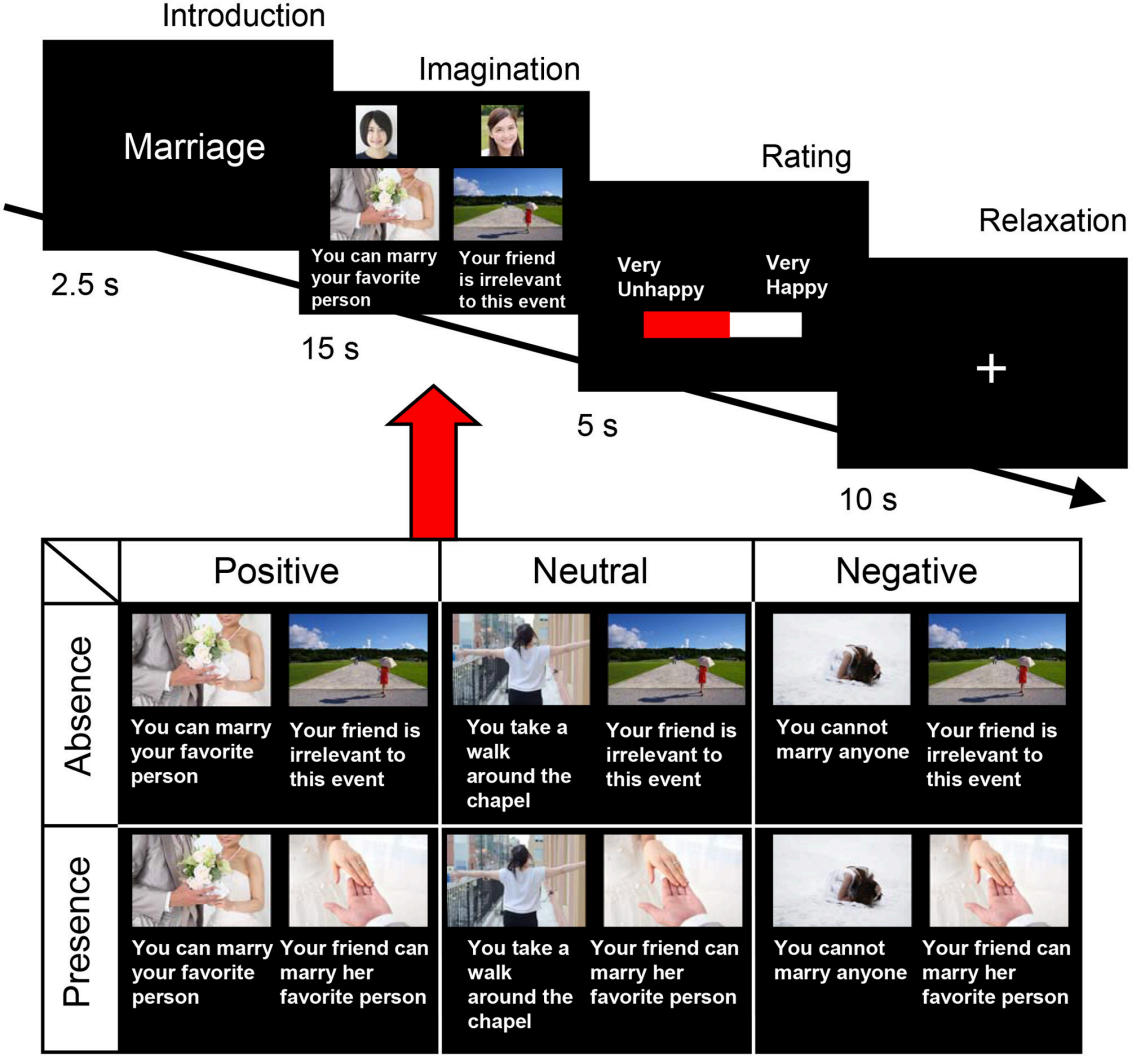
Sequence of events in each trial. Each trial consisted of the following sequence: (1) an introductory text phrase explaining the life event (2.5 s), (2) the imagination phase (15 s), (3) a rating phase (5 s), and (4) a relaxation phase (10 s). During the imagination phase, a picture was presented along with a statement pertaining to the outcome (positive/absence, neutral/absence, negative/absence, positive/presence, neutral/presence, or negative/presence). The example pictures representing each life event were actually used in the functional magnetic resonance imaging experiment in the present study. The photographs of participants were presented above the picture of each life event. Statements were written in Japanese for this task. We obtained copyright-free pictures representing each life event, as well as the sample face images used in this figure, from the Internet (http://www.photo-ac.com/).

The fMRI experiment was carried out using friend pairs. The participants were asked to enter the MRI gantry one by one, and to perform the happy-feelings evocation task (life event imagination task) depicted in Figure 1. These life events included several events that the participants experienced alone and several that they experienced with a friend. The events had three components: the occasion, the outcome, and the accompaniment. We selected 10 occasions based on our previous emotional event imagination task, which was shown to induce happy feelings (Matsunaga et al., 2016). As shown in Table 1, each occasion involved one of three outcomes (i.e., positive, neutral, or negative). We used these occasions to determine whether happiness-related empathy is invoked irrespective of one's own state. Participants imagined experiencing each outcome alone and with a happy friend (i.e., absence, presence). Therefore, a 3 (valences) × 2 (friend's presence/absence) within-subjects design was used. The experimental stimuli comprised 60 scenarios (i.e., 10 occasions, 3 outcomes, and 2 levels of accompaniment). We obtained photographs of the participants and copyright-free pictures representing each life event from the Internet (http://www.photo-ac.com). We presented the photographs of the participants and the event images on a display. The situation of the person performing the task was displayed on the left side and that of their friend was displayed on the right side (Figure 1).

**Table 1:** Hypothetical life events used in the functional magnetic resonance imaging task (10 occasions).

**Occasion**	**Outcome**	**Accompaniment**
Classroom	Your performance is excellent and you have been honored (positive). You read the newspaper in the classroom (neutral). Your performance is the worst (negative).	The performance of your friend is excellent and your friend is honored (presence).
Family	Your family feels happy (positive). You read a book alone in your room (neutral). Your family always has quarrels (negative).	Your friend's family feels happy (presence).
Club activity	You are the star of your club (positive). You drink tea in the clubhouse (neutral). You are the burden of your club (negative).	Your friend is the star of the club (presence).
Travel to Okinawa	You very much enjoy traveling (positive). You look at a building (neutral). You cannot return home because of a typhoon (negative).	Your friend very much enjoys traveling (presence).
Marriage	You can marry your favorite person (positive). You take a walk around the chapel (neutral). You cannot marry anyone (negative).	Your friend can marry her favorite person (presence).
Proposing to a romantic partner	The proposal is accepted (positive). The proposal is yet to be made (neutral). The proposal is rejected (negative).	The proposal of your friend is accepted (presence).
Lunch	You enjoy yourself with your friends (positive). You search for a restaurant (neutral). Your invitation is refused (negative).	Your friend enjoys herself with her friends (presence).
Job hunting	You receive an informal invitation from the company for which you want to work (positive). You chat away with someone (neutral). You receive no informal invitation from any company (negative).	Your friend receives an informal invitation from the company for which they want to work (presence).
Relationship with friends	You establish multiple truly trustful relationships with your friends (positive). You talk on the phone with someone (neutral). You cannot establish a truly trustful relationship with anyone (negative).	Your friend establishes multiple truly trustful multiple relationships with her friends (presence).
Relationship with romantic partner	You and your partner are in love with each other (positive). You and your partner use a personal computer (neutral). You break up with your partner (negative).	Your friend and her partner are in love with each other (presence).

*The outcomes comprise the participant's three situations (positive, neutral, and negative) and their friend's two situations (presence and absence). There are 60 outcomes in total. The situation of the participant's friend in the absence condition was prefaced by “Your friend is irrelevant to this event.” All statements in the task were written in Japanese*.

Each trial commenced with the presentation of a fixation cross for 20 s, after which the following sequence commenced: (1) an introductory text phrase, explaining the life event (2.5 s); (2) the imagination phase (15 s); (3) a rating phase (5 s); and (4) a relaxation phase (10 s). During the imagination phase, a picture was presented along with a statement pertaining to the outcome (positive/absence, neutral/absence, negative/absence, positive/presence, neutral/presence, or negative/presence), and the participants were asked to imagine how happy they would feel in these hypothetical situations. Following the picture presentation, the participants were allowed 5 s to rate their current level of happiness using a visual analog scale (VAS) labeled at the 0% (“very unhappy”), 50% (“neither happy nor unhappy”), and 100% (“very happy”) points of the scale. Subsequent to the rating, the participants had a 10-s period of rest, during which they looked at a fixation cross before the onset of the next trial. The experiment consisted of 60 trials. Five functional imaging runs (12 trials, about 7 min in total) were performed for each participant, and the 10 × 6 conditions were presented pseudo-randomly throughout the whole acquisition run. Participants received five training trials prior to the experiment to familiarize themselves with the procedure. The order of the six conditions was also counterbalanced across participants. The elevation of a participant's happy feelings by the presence of a happy friend was deemed indicative of happiness-related empathy.

#### Statistical analyses of behavioral data from the fMRI paradigm

Behavioral results are expressed as means ± standard errors of the mean (SEMs). The rating scores for happiness in each condition were compared using two-factor (valence of the event [positive, neutral, or negative] and presence of friends [absence or presence]) analysis of variance (ANOVA) followed by Bonferroni-corrected multiple comparisons.

#### fMRI data acquisition

Functional imaging was conducted using a 3-Tesla MRI scanner (Verio; Siemens Ltd., Erlangen, Germany). Each participant's head was immobilized within a 32-element phased-array head coil. Imaging was performed using an echo-planar imaging (EPI) gradient-echo sequence (echo time [TE] = 30 ms, repetition time [TR] = 2,500 ms, field of view [FOV] = 192 × 192 mm^2^, flip angle = 80°, matrix size = 64 × 64, 39 slices, slice thickness = 3 mm, total number of volumes = 168). A whole-brain, high-resolution T1-weighted anatomical magnetization-prepared rapid-acquisition gradient echo (MP-RAGE) MRI was also acquired for each participant (TE = 1.98 ms, TR = 1,800 ms, FOV = 256 × 256 mm^2^, flip angle = 9°, matrix size = 256 × 256 pixels, and slice thickness = 1 mm).

#### fMRI data preprocessing and analysis

We used Statistical Parametric Mapping (SPM) software (SPM12 revision 6225; The Wellcome Department of Cognitive Neurology, London, UK) implemented in MATLAB 2014b (MathWorks Inc., Natick, MA) to analyze the functional images. The first four volumes of each fMRI run were discarded due to unsteady magnetization. After all of the volumes were realigned, differences in slice timing within each image volume were corrected. The reference image was at the center of the volume. The whole-brain 3D MP-RAGE volume was co-registered to the EPI volumes and normalized to the Montréal Neurological Institute T1 image template (ICBM152; Evans et al., 1994) using a non-linear basis function. Subsequently, normalization parameters were applied to all of the EPI volumes. The normalized EPI images were then spatially smoothed in three dimensions using an 8-mm full-width at half-maximum Gaussian kernel. After the realignment process, we checked the data for head movements >3 mm during the experimental run. Task-related activation was evaluated on a voxel-by-voxel basis using the general linear model at the individual level to generate contrast images. The introduction (2.5 s), imagination (15 s), and rating phases (5 s) were separately modeled using a block design convolved with the canonical hemodynamic response. The introduction and rating phases were considered covariates of no interest in order to partial out their contribution to brain activation in the single participant analyses. Using contrast images related to the imagination phases of the six conditions (positive/absence, neutral/absence, negative/absence, positive/presence, neutral/presence, and negative/presence; Figure 1), we conducted a random-effects analysis at the group level (Friston, 2007) using a full factorial design. This group analysis approach, typically referred to as the *Summary Statistics* approach, is based on a two-level strategy (Stephan et al., 2006; Monti, 2011). We used the following hierarchical two-level linear model:

$$\begin{array}{ll} Y & =X\beta +\varepsilon \;\left(1\text{st level},\text{ fixed effects}\right)\\ \beta =X_{G}\beta_{\text{G}}+\varepsilon_{\text{G}}\;\left(2\text{nd level},\text{ random effects}\right) \end{array}$$

In the above formula, “*X*” is the single-subject design matrix, “*X*_*G*_” is a group-level matrix, “ε” is the individual-specific error, and “ε_G_” is the group-specific error. The statistical threshold was set at an uncorrected *p* < 0.001 at the voxel level and a family-wise error (FWE)-corrected *p* < 0.05 at the cluster level (whole brain). The plot function in SPM12 was used to generate the plots of parameter estimates and 90% confidence intervals (CIs) at the regions of interest (ROIs) (mentalizing/theory-of-mind network), such as voxels [−2, 58, 20] (mPFC), [0, −60, 32] (precuneus), [−56, −10, −20] (temporal pole), and [−48, −64, 32] (TPJ). These parameter estimates were then extracted from MATLAB and used to create bar graphs.

Furthermore, neural responses associated with individual happiness ratings were assessed using a parametric modulation analysis, wherein the participant's ratings associated with each event from all conditions were entered as covariates in the individual-level analysis. We then conducted a random-effects analysis at the group level using a one-sample *t*-test design. The statistical threshold was set at an uncorrected *p* < 0.001 at the voxel level and an FWE-corrected *p* < 0.05 at the cluster level (whole brain). A statistical parametric map was masked with an atlas in SPM12 (Labels_neuromorphometrics: anterior cingulate gyrus, temporal pole, superior temporal gyrus, and precuneus). We analyzed the correlations between activities in the mentalizing/theory-of-mind network and changes in happiness rating scores for each condition in the fMRI task. We conducted a group analysis with a multiple regression design using subtracted rating scores for happiness and contrast images (presence – absence) for each valence of the imagined event (positive, neutral, and negative). Based on the result of the full factorial analysis (Table 2), ROI analysis was conducted using small volume correction (SVC; a 15-mm radius sphere for the peak coordinates of mPFC, precuneus, TPJ, and temporal pole, which are shown in Table 2). The statistical threshold was set at an uncorrected *p* < 0.001 at the voxel level and an FWE-corrected *p* < 0.05 at the cluster level (ROI). The plot function in SPM12 was used to generate a plot of the adjusted contrast estimates at voxels [58, 2, −22] (temporal pole) and [52, −62, 32] (TPJ). These parameter estimates were extracted from MATLAB and used to create a scatterplot.

**Table 2:** Neural regions that were more active in the presence conditions than in absence conditions.

**Region**	**Cluster size**	**MNI Coordinates**			***t*-value**	**Cluster *p*-value (FWE- corrected)**
		**x**	**y**	**z**		
Medial prefrontal cortex	1,670	−2	58	20	5.68	<0.01
		−4	52	−14	4.08	
		18	36	54	4.03	
Precuneus	1,212	0	−60	32	5.26	<0.01
		4	−48	10	3.52	
Left temporal pole/middle temporal gyrus	889	−56	−10	−20	5.05	<0.01
		−64	−8	−16	4.72	
		−52	8	−30	4.51	
Left temporoparietal junction	413	−48	−64	32	4.95	0.011
		−42	−52	28	3.35	
Right temporal pole/middle temporal gyrus	571	34	20	−18	4.27	<0.01
		52	12	−30	4.17	
		62	−4	−24	4.08	
Right temporoparietal junction	313	52	−60	28	4.26	0.032
		58	−58	22	4.07	
Caudate/septal area/thalamus	1,796	−12	12	8	4.89	<0.01
		8	−4	8	4.57	
		20	8	4	4.46	
Visual cortex	277	12	−82	4	4.40	0.048
		16	−70	2	3.61	
Cerebellum	485	0	−52	−42	5.42	<0.01
	374	−28	−72	−32	3.93	0.016
		−30	−82	−32	3.71	
		−24	−78	−38	3.58	

*Neural regions that were more active in the presence conditions than in absence conditions regardless of the valence of the hypothetical situation (main effect of the presence of friends). FWE, Family-wise error. We used SPM12 to calculate t statistics and the threshold, which was t = 3.12. Voxel size: 3 × 3 × 3 mm^3^*.

#### Genotyping and analysis

Genomic DNA was extracted from nail samples from the participants using ISOHAIR kits (Nippon Gene Co., Ltd., Tokyo, Japan). The SNP marker for rs6311 was genotyped using the TaqMan® SNP Genotyping Assay (Thermo Fisher Scientific Inc., Waltham, MA), which was functionally tested by Thermo Fisher Scientific Inc. and available on demand. Each SNP assay contains forward and reverse polymerase chain-reaction (PCR) primers as well as two allele-specific probes conjugated with either VIC or FAM fluorescent marker. Each PCR mixture consisted of DNA templates, the SNP-specific genotyping assay, and Taqman genotype master mix (Thermo Fisher Scientific Inc.). All PCRs and allelic discrimination reactions were performed on the StepOne Plus™ Real-Time PCR System (Thermo Fisher Scientific Inc.). The genotype distribution for the *HTR2A* variants was as follows: 10 AA, 31 GA, and 12 GG. The *HTR2A* genotype distribution for the entire population was similar to that reported in previous studies including East Asian samples (Lee and Ham, 2008; Dressler et al., 2009). The demographic data (age and BMI) were compared using one-way ANOVAs (*HTR2A* genotypes), and subtracted rating scores for happiness (presence – absence) for each valence of the imagined event (positive, neutral, and negative) were compared using a 3 (valences) × 3 (*HTR2A* genotypes) ANOVA followed by Bonferroni-corrected multiple comparisons.

Because we excluded three participants from the analysis (excessive body movement), we analyzed MRI images from 50 participants (10 AA, 29 GA, and 11 GG). Using the contrast images derived from images captured during the imagination phase (neutral/presence – neutral/absence), we conducted a random-effects analysis at the group level (Friston, 2007) using a one-way ANOVA design. We conducted a subtraction analysis (GG + AG > AA) to reveal the brain regions that were strongly activated in G carriers, compared to those with the AA genotype. Based on the results of the full factorial analysis (Table 2), ROI analysis was conducted using SVC (a 10-mm radius sphere for the peak coordinates of mPFC, precuneus, TPJ, and temporal pole, which are shown in Table 2). The statistical significance threshold was set at an uncorrected *p* < 0.001 at the voxel level and an FWE-corrected *p* < 0.05 at the cluster level (ROI). The plot function in SPM12 was used to generate the plots of contrast estimates and 90% CIs at the voxel [56, 10, −26] (temporal pole). These contrast estimates were then extracted from MATLAB and used to create bar graphs.

### Questionnaire

#### Participants

We recruited 206 healthy male and female volunteers (age range: 18–23 years, mean age: 19.2 years) following the study's approval by the Ethics Committee of Kobe University (approval number: 2014-10) to study sex differences. All participants provided written informed consent in accordance with the Declaration of Helsinki. All participants were Japanese undergraduate students at Kobe University.

We tried to collect as much data as possible given the constraints of our research funding. A statistical power analysis was conducted using G^*^Power version 3.1.9.2 (Faul et al., 2007). We assumed that the effect size of this study would be equivalent to that observed in the study by Lebe et al. (2013), who found a significant association between depressive symptoms and *HTR2A* genotypes. *A priori* power analysis was used to estimate the necessary sample size for this study as *n* = 159 (ANOVA: fixed effects, omnibus, one-way, *F*-tests; effect size = 0.25; alpha error = 0.05; 1-beta error = 0.8; number of groups = 3).

The mean BMI of all participants was 20.6 (range: 15.6–32.7). There were significant differences in age (women: 19.0, men: 19.5, *p* < 0.01) and BMI (women: 20.2, men: 20.9, *p* < 0.05) between men and women. Because no participants smoked and only 26 participants consumed alcohol, we discounted these potential confounding factors in subsequent analyses.

#### Genotyping

*HTR2A* genotype was determined using a similar method to that utilized in the fMRI experiment. The genotype distribution of *HTR2A* was as follows. Women: 30 AA, 59 GA, and 18 GG; men: 28 AA, 45 GA, and 26 GG. The *HTR2A* genotype distribution for the entire population was similar to that observed in the fMRI experiment, and to those reported in previous studies including East Asian samples (Lee and Ham, 2008; Dressler et al., 2009).

#### Content of the questionnaire

Based on results of our fMRI experiment, we compared happy feelings between the presence and absence conditions in the neutral (self-valence) event. We selected one occasion (personal relationship) from the 10 occasions used in the fMRI experiment. The questionnaire contained two items. The participants were asked to evaluate their happy feelings on a 7-point Likert scale (1: extremely unhappy; 2: very unhappy; 3: a little unhappy; 4: neither; 5: a little happy; 6: very happy; 7: extremely happy) when they encountered the following two situations: “You had no change in your interpersonal relationship (your friend is irrelevant to this event)” (neutral/absence condition), and “You had no change in your interpersonal relationship, but your friend developed a bond with someone he/she can trust” (neutral/presence condition).

#### Statistical analyses of questionnaire results

We subtracted the rating score of the first question (neutral/absence) from that of the second question (neutral/presence) and compared the difference scores (happiness-related empathy) between *HTR2A* genotypes using a 3 (*HTR2A* genotypes) × 2 (sex) ANOVA followed by Bonferroni-corrected multiple comparisons. Furthermore, in order to remove the influences of several confounding factors, such as age, sex, and BMI, from the effects of *HTR2A* on happiness-related empathy, we used the following regression model:

$$\begin{array}{l} Y=\beta_{0}+\beta_{1}\text{H}+\beta_{2}\text{S}+\beta_{3}\text{A}+\beta_{4}\text{B}+\varepsilon \end{array}$$

In the above formula, “H” is a matrix of variables to control for the *HTR2A* genotype, whereby H = 2 if the participant's genotype is GG, H = 1 if the genotype is AG, and H = 0 if the genotype is AA. “S” is a matrix of variables used to control for sex (S = 0 if the subject is male and S = 1 if the subject is female). “A” is a matrix of variables used to control for age, and “B” is a matrix of variables used to control for BMI, while “ε” is the individual-specific error.

#### Evaluation of subjective happiness level

To assess subjective levels of happiness, participants completed the Japanese version of the Subjective Happiness Scale (JSHS; Shimai et al., 2004; Matsunaga et al., 2011a,b), which is based on the Subjective Happiness Scale (SHS) originally developed by Lyubomirsky and Lepper (1999). The SHS exhibits excellent psychometric properties, such as high internal consistency, a unitary structure, and stability over time (Lyubomirsky and Lepper, 1999). Therefore, the SHS is a widely used psychometric tool for evaluating subjective happiness levels (Shimai et al., 2004; Matsunaga et al., 2011a,b; Zhang et al., 2013). The JSHS subjectively assesses whether a person is happy or unhappy, as well as his or her positive personal traits. Each item is answered on a 7-point Likert scale. We asked participants to circle the point on the scale that they felt described them most accurately. The items were as follows: (1) “In general, I consider myself…” 1 (not a very happy person) to 7 (a very happy person); (2) “Compared to most of my peers, I consider myself…” 1 (less happy) to 7 (more happy); (3) “Some people are generally very happy. They enjoy life regardless of what is going on, getting the most out of everything. To what extent does this characterization describe you?” 1 (not at all) to 7 (a great deal); and (4) “Some people are generally not very happy. Although they are not depressed, they never seem as happy as they might be. To what extent does this characterization describe you?” 1 (not at all) to 7 (a great deal). The internal consistency, test-retest reliability, and convergent discriminant validity of the JSHS have previously been confirmed (Lyubomirsky and Lepper, 1999; Shimai et al., 2004; Matsunaga et al., 2011a,b). Cronbach's alpha for the JSHS was 0.81 in the present study.

## Results

### Manipulation check of the present fMRI task

**Figure 2:**
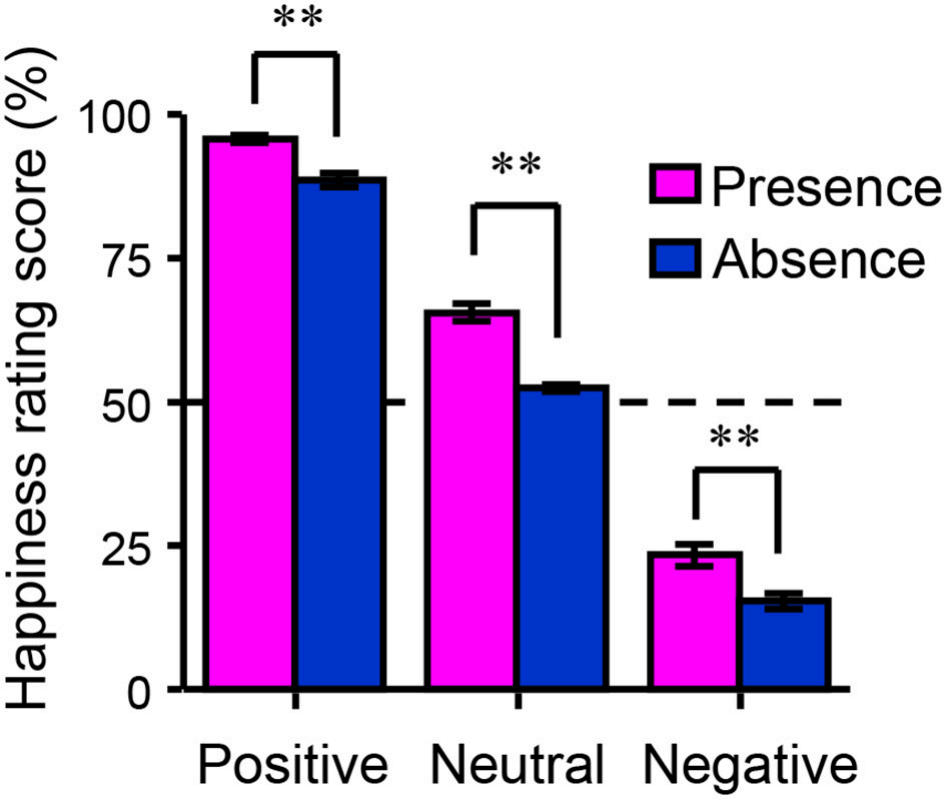
Behavioral data from the functional magnetic resonance imaging experiment. The bar graph shows happiness rating scores as a function of the participants' emotional valences (positive, neutral, or negative) and the presence of a happy friend (presence or absence). Each column and the error bars represent means ± standard errors of the mean of the score (*n* = 53). ^**^p < 0.01 vs. absence condition.

**Figure 3:**
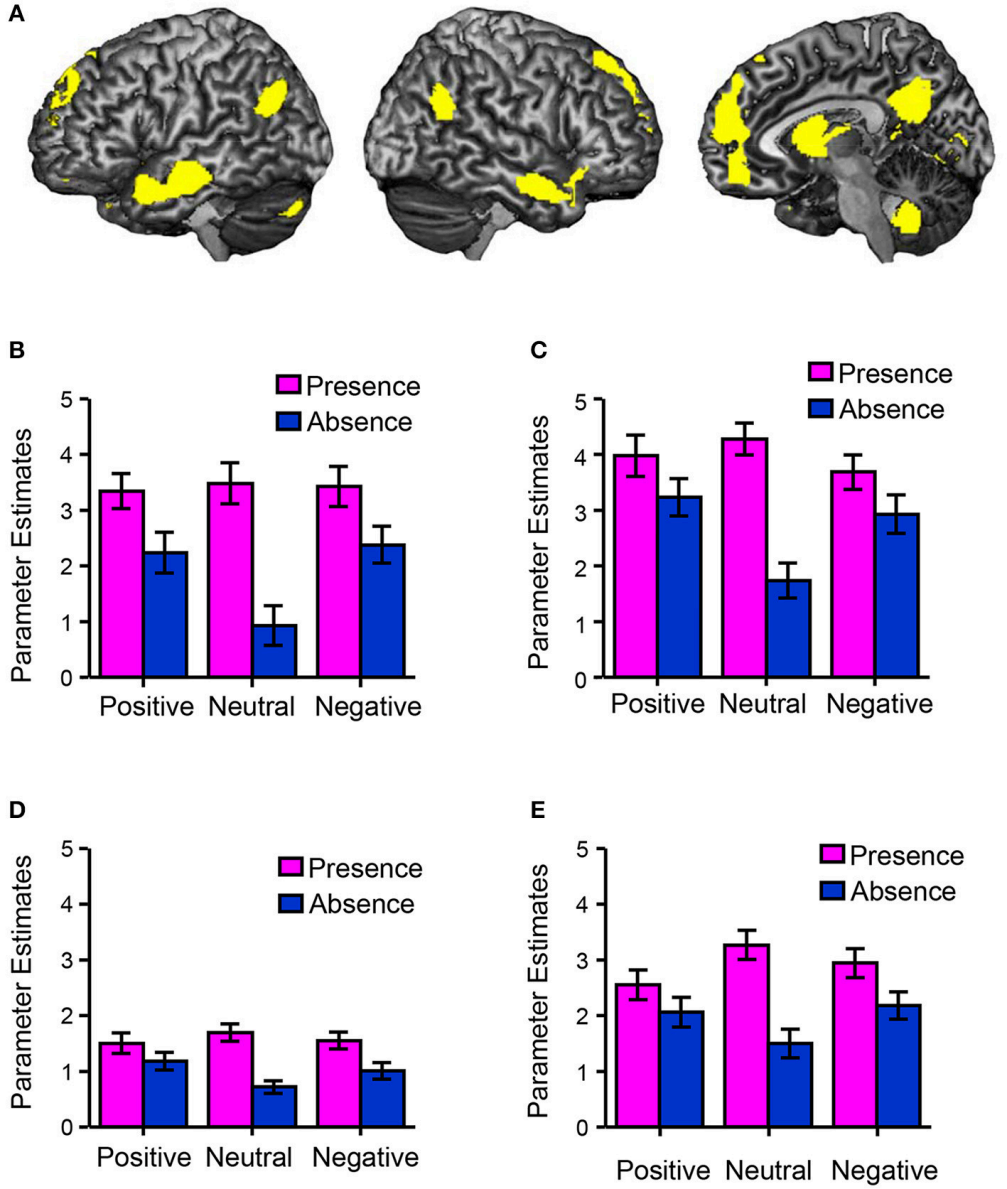
Brain regions associated with happiness-related empathy. **(A)** A statistical parametric map illustrating the cluster (yellow) that was significantly activated in the presence condition when compared to the absence condition (main effect of the presence of friends). Statistical significance thresholds were set at *p* < 0.001 (uncorrected) at the voxel level and *p* < 0.05 (familywise-error-corrected, whole brain) at the cluster level (*n* = 50). The bar graphs show the brain activities (parameter estimates) at the regions of interest (mentalizing/theory-of-mind network), such as voxels [−2, 58, 20] (mPFC) **(B)**, [0, −60, 32] (precuneus) **(C)**, [−56, −10, −20] (temporal pole) **(D)**, and [−48, −64, 32] (TPJ) **(E)**.

Figure 2 shows the happiness ratings for the six conditions used in the present study (positive/absence, neutral/absence, negative/absence, positive/presence, neutral/presence, and negative/presence). A 3 (valences) × 2 (friend's presence or absence) ANOVA revealed a significant main effect of the presence of a friend on happiness rating scores [*F*_(1, 156)_ = 139.06, *p* < 0.01, η^2^_*p*_ = 0.47] and a significant interaction between the valence of the event and the presence of a friend [*F*_(2, 156)_ = 5.30, *p* < 0.01, η^2^_*p*_ = 0.06]. A multiple comparisons test indicated that happiness rating scores in the presence condition were significantly higher than those in the absence condition (*p* < 0.01) regardless of the valence. Happiness rating scores in the positive condition were significantly higher than those in the negative (*p* < 0.01) and neutral conditions (*p* < 0.01) regardless of the presence of friends. We then studied the main effect of the presence of friends on brain activity using a random-effects analysis at the group level with a full factorial design. As shown in Figure 3A (see also Table 2), the mPFC, precuneus, temporal pole, TPJ, caudate/septal area/thalamus, visual cortex, and cerebellum exhibited significantly higher activation in the presence condition when compared to the absence condition (*p* < 0.05, FWE-corrected [whole brain]). Bar graphs showing parameter estimates for each brain region indicate that the mentalizing/theory-of-mind network [mPFC (Figure 3B), precuneus (Figure 3C), temporal pole (Figure 3D), and TPJ (Figure 3E)] were more activated in the presence condition than in the absence condition. We subsequently assessed interactions between valence and friend's presence/absence for the fMRI data, as we did for the happiness ratings. However, no significant interaction between the valence of the event and the presence of a friend was observed.

### Association between brain activity and changes in happiness rating scores during the fMRI task

**Figure 4:**
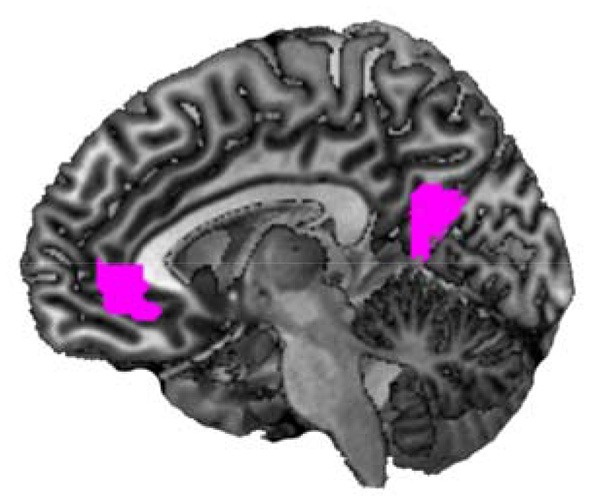
Parametric modulation of brain activity patterns using happiness rating scores. A statistical parametric map illustrating the cluster (violet) of brain areas showing parametric modulation of activity associated with changes in happiness rating scores, including all six conditions (*n* = 50).

**Figure 5:**
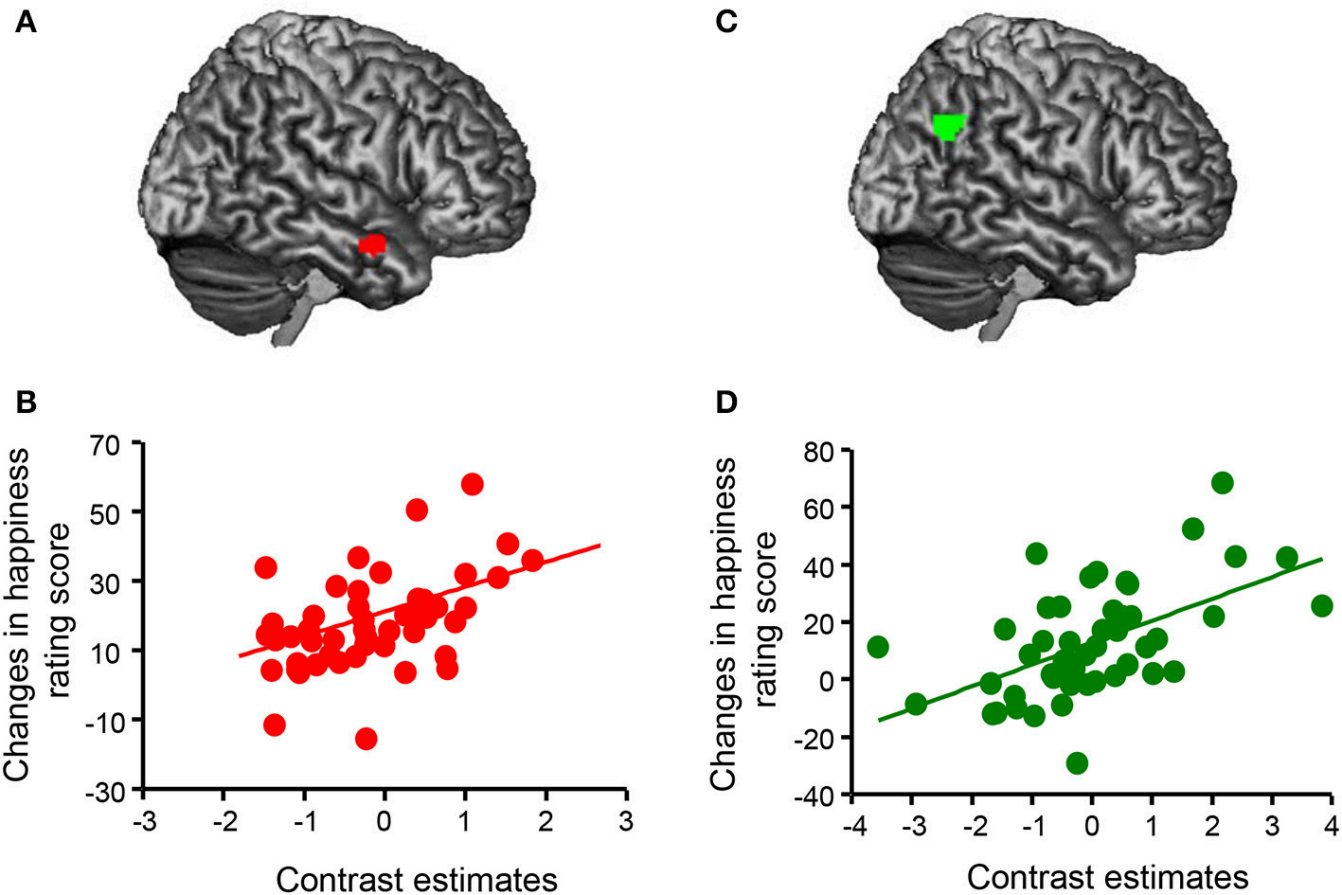
Associations between brain activity and changes in happiness rating scores. **(A)** A statistical parametric map illustrating the cluster (red) that was significantly correlated with subtracted happiness rating scores (presence – absence) in the neutral condition. Statistical significance thresholds were set at *p* < 0.001 (uncorrected) at the voxel level and *p* < 0.05 (familywise-error [FWE]-corrected; small volume correction [SVC], a 15-mm radius sphere at the voxel [52, 12, −30], right temporal pole) at the cluster level. **(B)** The scatterplot shows the positive correlation between adjusted contrast estimates at [58, 2, −22] and changes in happiness rating scores (presence – absence) in the neutral condition (*n* = 50). **(C)** A statistical parametric map illustrating the cluster (green) that was significantly correlated with subtracted happiness rating scores (presence – absence) in the negative condition. Statistical significance thresholds were set at *p* < 0.001 (uncorrected) at the voxel level and *p* < 0.05 (FWE-corrected; SVC, a 15-mm radius sphere at the voxel [52, −60, 28], right temporoparietal junction) at the cluster level. **(D)** The scatterplot shows the positive correlation between adjusted contrast estimates at [52, −62, 32] and changes in happiness rating scores (presence – absence) in the negative condition (*n* = 50).

First, we determined brain areas showing parametric modulation of activity in the mentalizing/theory-of-mind network (mPFC, precuneus, temporal pole, and TPJ) associated with changes in happiness rating scores for all conditions (Figure 4). We found that activations of the mPFC (peak coordinates: x = −8, y = 42, z = −4; *p* < 0.05, FWE-corrected [whole brain]; cluster size = 394; *t* = 7.18) and precuneus (peak coordinates: x = −10, y = −56, z = 14; *p* < 0.05, FWE-corrected [whole brain]; cluster size = 378; *t* = 5.95) were positively associated with happy feelings. Second, we analyzed the correlations between activity in the mentalizing/theory-of-mind network and changes in happiness rating scores for each condition in the fMRI task. Regression analysis indicated that the subtracted happiness rating score (presence – absence) was positively correlated with the activity of the right temporal pole in the neutral condition (peak coordinates: x = 58, y = 2, z = −22; *p* < 0.05, FWE-corrected [ROI]; cluster size = 18; *t* = 4.52; Figure 5A). The scatterplot in Figure 5B shows the positive correlation between the adjusted contrast estimates at [58, 2, −22] and the subtracted happiness rating scores. There were no associations between other brain regions and changes in happiness rating scores at this threshold. Furthermore, regression analysis indicated that subtracted happiness rating scores were positively correlated with the activity of the right TPJ in the negative condition (peak coordinates: x = 52, y = −62, z = 32; *p* < 0.05, FWE-corrected [ROI]; cluster size = 100; *t* = 4.54; Figure 5C). The scatterplot in Figure 5D shows the positive correlation between the adjusted contrast estimates at [52, −62, 32] and the subtracted happiness rating scores. There were no associations between other brain regions and changes in happiness rating score at this threshold. We did not find significant correlations between any brain regions and changes in happiness scores in the positive condition.

### Association between HTR2A and the effect of sharing happiness in the fMRI task

**Figure 6:**
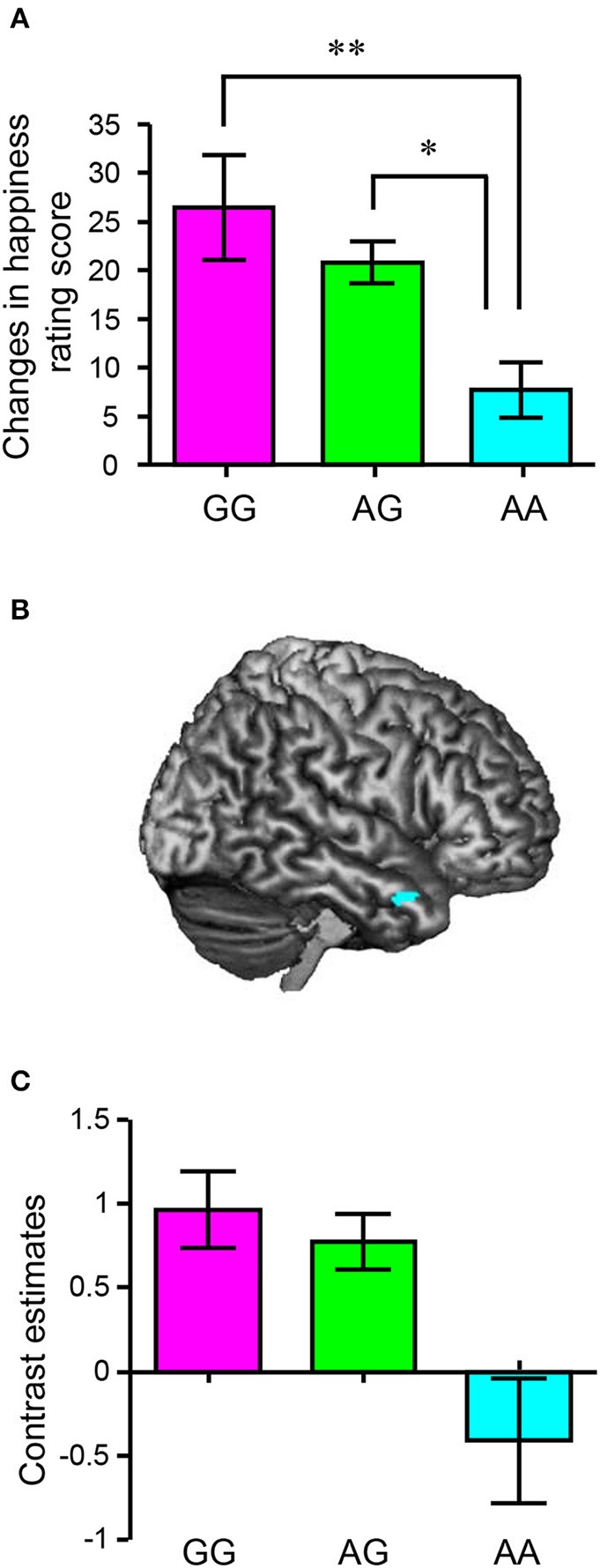
Associations between *HTR2A* polymorphisms and happiness-related empathy. **(A)** Mean difference score (neutral/presence – neutral/absence) as a function of *HTR2A* genotype (original visual analog scale score: 0–100). Each column and the error bars represent means ± standard errors of the mean of the rating score. ^**^p < 0.01 and ^*^p < 0.05 vs. AA genotype (*n* = 10 AA, 31 GA, and 12 GG). **(B)** A statistical parametric map illustrating the cluster (cyan) that was significantly activated in G carriers, compared to individuals with the AA genotype using contrast images (neutral/presence – neutral/absence). The statistical significance threshold for the analysis was set at *p* < 0.001 (uncorrected) at the voxel level and *p* < 0.05 (familywise-error-corrected; small volume correction, a 10-mm radius sphere around the voxel [52, 12, −30], right temporal pole) at the cluster level (*n* = 10 AA, 29 GA, and 11 GG). **(C)** A bar graph indicating the differences in the contrast estimates at [56, 10, −26].

We subsequently assessed the association between *HTR2A* and the effect of sharing happiness. In the fMRI experiment, we divided the participants into three groups based on their *HTR2A* genotypes (GG, AG, and AA) and compared changes in happiness rating score (presence – absence) in each valence condition (positive, neutral, and negative) between *HTR2A* genotypes. There were no significant differences in age (AA: 20.4, AG: 21.0, and GG: 20.8) or BMI (AA: 20.3, AG: 20.2, and GG: 19.7) between individuals with different *HTR2A* genotypes, as revealed by a one-way ANOVA, although the mean values of BMI suggested a trend for co-variation with *HTR2A* genotype. Thus, we discounted these confounding factors in subsequent analyses. Although a 3 (valences) × 3 (*HTR2A* genotypes) ANOVA did not reveal a significant interaction between the valence of the event and *HTR2A* genotype [*F*_(2, 150)_ = 1.50, *p* = 0.20, η^*2*^_p_ = 0.04], a multiple comparisons test indicated that the subtracted happiness rating scores in individuals with the AA genotype were significantly lower than those in individuals with the AG (*p* < 0.05) and GG (*p* < 0.01) genotypes in the neutral condition (Figure 6A and Table 3). The subtracted happiness rating scores were not significantly different between individuals with different *HTR2A* genotypes in either the positive or the negative condition (Table 3). We found that the activity of the right temporal pole was significantly lower in participants with the AA genotype than in those with the AG and GG genotypes in the neutral condition (peak coordinates: x = 56, y = 10, z = −26; *p* < 0.05, FWE-corrected [ROI]; cluster size = 15; *t* = 3.79; Figure 6B). This is also indicated in the bar graph showing contrast estimates for this region (Figure 6C).

**Table 3:** Mean happiness difference score (presence – absence) as a function of event valence (positive, neutral, or negative) and HTR2A genotype in the functional magnetic resonance imaging experiment.

	***HTR2A*** **genotype**		
	**GG**	**AG**	**AA**
Positive	10.1 ± 3.5	10.4 ± 1.4	12.4 ± 4.6
Neutral	26.4 ± 5.3^**^	20.7 ± 2.1^*^	7.6 ± 2.8
Negative	14.5 ± 5.6	13.2 ± 3.1	5.2 ± 5.6

*Results are expressed as means ± standard errors of the mean*.

* *p < 0.05*,

** *p < 0.01 vs. AA genotype. Happiness difference scores for each valence of the imagined event (positive, neutral, and negative) were compared using Bonferroni-corrected multiple comparisons*.

### Validation of the association between HTR2A and the effect of sharing happiness using the questionnaire

**Figure 7:**
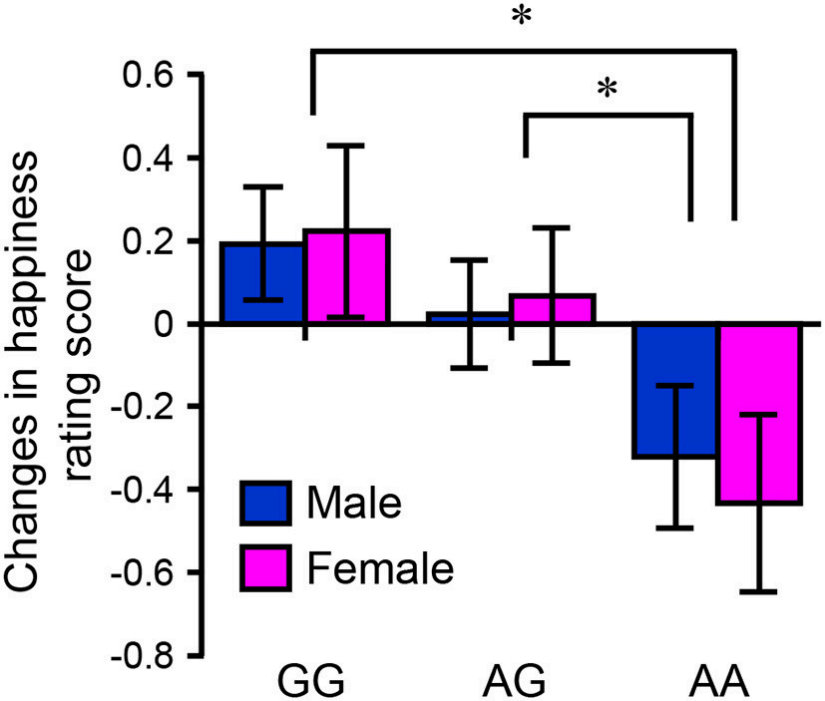
Results of the questionnaire experiment. The bar graph indicates the mean difference score (neutral/presence – neutral/absence) as a function of sex and *HTR2A* genotype (original evaluation score: 1–7) (women: 30 AA, 59 GA, and 18 GG; men: 28 AA, 45 GA, and 26 GG). Each column and error bar represents the mean ± standard error of the mean of the rating score. ^*^p < 0.05 vs. AA genotype.

The questionnaire included two situations similar to those used in the fMRI study (neutral/absence and neutral/presence). The subtracted rating scores were compared between individuals with different *HTR2A* genotypes. Although there was no main effect of *HTR2A* on age, there was a main effect of *HTR2A* on BMI [*F*_(2, 200)_ = 3.21, *p* < 0.05]. The mean BMI in the AA genotype group was significantly higher than that in the GG genotype group (*p* < 0.05 [Bonferroni-corrected multiple comparisons]; AA: 21.1 ± 0.41 [SEM], AG: 20.6 ± 0.34, GG: 19.9 ± 0.47). A two-factor (*HTR2A* and sex) ANOVA revealed a significant main effect of *HTR2A* on rating score [*F*_(2, 200)_ = 4.70, *p* < 0.01, η^*2*^_p_ = 0.45], and a multiple comparisons test indicated that the subtracted happiness rating score in the AA genotype group was significantly lower than those in the AG (*p* < 0.05) and GG (*p* < 0.05) genotype groups (Figure 7). Table 4 shows the results of the multiple regression analysis, which tested the hypothesis that variations in *HTR2A* are associated with the subtracted happiness rating score even after controlling for the potentially confounding variables of age, sex, and BMI. This regression model was statistically significant [*F*_(4, 201)_ = 2.47, *p* < 0.05] and confirmed that individuals with G polymorphisms in *HTR2A* are significantly more susceptible to a friend's positive emotions than those with the AA genotype (*p* < 0.01).

**Table 4:** Results from the regression analysis examining the association between *HTR2A* and subtracted happiness rating score.

**Predictor variables**	**β**	***t***	***P*-value**
***HTR2A*** **G**	**0.198**	**2.814**	**<0.01**
Sex	0.012	0.168	0.867
Age	0.074	1.048	0.296
BMI	−0.010	−0.138	0.890
*N*	206		
Adjusted *R*^2^	0.028		

*All predictor variables were included in the regression analysis. Boldface indicates statistically significant variables. β, Standardized beta coefficient; BMI, body mass index*.

We also compared the participants' subjective happiness levels according to *HTR2A* genotype using the JSHS because, as discussed in the Introduction, susceptibility to others' happiness might be associated with subjective happiness. The mean JSHS scores were as follows: women, AA: 4.57 ± 0.16 (SEM), GA: 4.93 ± 0.12, and GG: 4.94 ± 0.23; men, AA: 4.66 ± 0.16, GA: 4.59 ± 0.16, and GG: 4.50 ± 0.20. Although the mean JSHS score for female G carriers was higher than that for female individuals with the AA genotype, statistical analysis using Bonferroni-corrected multiple comparisons did not reveal a significant difference between these groups.

## Discussion

### Neural correlates of the empathic modulation of happiness

We first tested whether participants' happiness was influenced by the presence of a companion. The fMRI results indicated that the participants rated their feelings more positively when their friends experienced positive events, regardless of the participant's situation (positive, neutral, or negative; Figure 2). The mPFC, precuneus, superior temporal sulcus, TPJ, caudate/septal area/thalamus, visual cortex, and cerebellum were significantly more active in the presence condition compared to the absence condition (Figure 3A). These brain regions include those previously reported as correlated with happiness-related empathy (mentalizing/theory-of-mind network; Morelli et al., 2014). Thus, the present fMRI task induced happiness-related empathy, which was invoked irrespective of the participant's own state. Further, the neural correlates of happiness-related empathy were located in the mentalizing/theory-of-mind network.

Subsequently, in order to study the association between changes in self-reported happy feelings and brain activity, we investigated the associations between behavioral data and brain activity. First, parametric modulation analysis including all conditions revealed that brain activity in the aforementioned regions associated with happiness-related empathy (mPFC and precuneus) were positively correlated with self-reported happy feelings (Figure 4). Previous studies reported associations between subjective happiness and the mPFC or precuneus (Sato et al., 2015; Matsunaga et al., 2016) that are consistent with our findings, and which may indicate that others' happy feelings modulate the participant's happy feelings. Second, regression analysis indicated that activity in the mentalizing/theory-of-mind network was associated with happiness rating scores in each condition. Changes in happiness rating scores were positively correlated with the activity of the right temporal pole in the neutral condition and the activity of the right TPJ in the negative condition (Figure 5). The temporal pole is commonly considered to be involved in encoding and representing social knowledge, and has a critical role in person memory (Olson et al., 2013). The right part of the temporal pole may be associated with feelings of familiarity and retrieval of biographical information (Olson et al., 2013). Thus, the association between increases in happy feelings and right temporal pole activity in the neutral condition might indicate that the more strongly the participants imagined the happy faces of their familiar friend, the more happy they felt. The reason that this correlation was observed only in the neutral condition may be that the participant could imagine only the happy face of a friend without having to imagine other competing factors, such as the participant's emotional state and face. The TPJ is also commonly considered to be a key brain region involved in social cognition. A previous neuroimaging study indicated that the right TPJ (supramarginal gyrus) is associated with the ability to understand others' feelings without being misled by one's own emotions. This is also known as the ability to overcome the emotional egocentricity bias (self-other distinction; Silani et al., 2013). That is, if right TPJ activity is relatively weak, participants may have rated their emotional states more negatively in the negative condition (incongruent situation; self: negative, friend: positive). The reason that the positive correlation between right TPJ activity and self-reported happy feelings was not observed in other conditions may be that the other conditions did not represent an incongruent situation. In contrast, there were no positive correlations between brain activity in any region and self-reported happy feelings in the positive condition. These effects may have been too small to detect, as the participant's situation was the same as the friend's situation in this condition.

### Association between HTR2A and happiness-related empathy

As expected, the fMRI experiment indicated that *HTR2A* is associated with happiness-related empathy. Differences in the happiness rating scores (neutral/presence – neutral/absence) were significantly less in individuals with the *HTR2A* AA genotype than in G allele carriers (Figure 6). Individuals with the *HTR2A* AA genotype felt less happy than the G allele carriers when their friends were in a happy situation. The fMRI experiment also indicated that the activity of the right temporal pole was significantly lower in individuals with the *HTR2A* AA genotype than it was in the G carriers when they imagined their friends experiencing positive events. The association between *HTR2A* and happiness-related empathy was also validated by our questionnaire, which indicated that the subtracted happiness rating score (neutral/presence – neutral/absence) was lower in individuals with the *HTR2A* AA genotype than in G carriers, regardless of sex (Figure 7). Multiple regression analysis, which controlled for age, sex, and BMI, showed that polymorphisms of the *HTR2A* gene were significantly associated with increased happiness rating scores (Table 4).

A previous study reported an association between BMI and serotonin 2A receptor expression in the human brain (Erritzoe et al., 2009). Although mean BMI (AA: 20.4, AG: 20.2, and GG: 19.7) was not significantly different between individuals with different *HTR2A* genotypes in our fMRI experiment, the mean BMI of participants with the AA genotype was significantly higher than that of individuals with the GG genotype (AA: 21.1, AG: 20.6, and GG: 19.9) in our questionnaire study. Based on the results of Erritzoe et al. (2009), the association between BMI and *HTR2A* genotype in our questionnaire experiment suggests that individuals with the AA genotype have high cerebral serotonin 2A receptor density because of increased body weight. However, the regression model indicated that the association between *HTR2A* polymorphism and empathy for happiness, even when controlling for BMI, suggests that the effect of genetic polymorphism on happiness-related empathy is remarkable regardless of the effect of weight gain on serotonin 2A receptor expression.

In addition, a recent PET neuroimaging study of the distribution of the serotonin 2A receptor in the human brain found its expression to be high in the temporal lobe (Savli et al., 2012). Thus, *HTR2A* may modulate the empathy-related functions of the temporal lobe, and specifically those of the temporal pole.

Our fMRI experiment also indicated that *HTR2A* did not influence the modulation of happiness in the positive or negative conditions (Table 3), although happiness-related empathy was invoked in both positive and negative conditions (Figure 2). This result suggests that positive and negative conditions may have evoked different emotional responses than the neutral condition. Happiness may be “transmitted” to someone in a neutral mood. This phenomenon may be conceptualized as emotional contagion, which refers to experiencing the same emotional conditions as those of others, without being aware of the fact (Hatfield et al., 1993). Previous studies have suggested that the right temporal pole plays a critical role in emotional contagion (Hillis, 2014), and our regression analysis revealed an association between happy feelings and right temporal pole activity in the neutral condition. Therefore, emotional contagion might have been strongly evoked in the neutral condition. In contrast, the situation wherein one's happiness is enhanced by a friend's happiness might be better conceptualized as the empathic modulation of happy feelings. Finally, the situation wherein one's negative feelings are alleviated by a friend's happiness may be more closely related to emotion regulation (Gross and John, 2003). Such emotional modulation and/or emotion regulation may be associated with cognitive transformation of the emotional experience (i.e., reappraisal), which alters the trajectory of an unfolding emotional process (Ochsner et al., 2004). Previous studies have suggested that the TPJ is associated with interpersonal emotion regulation (Grecucci et al., 2013), and our regression analysis revealed an association between happy feelings and TPJ activity in the negative condition. Therefore, emotion regulation might have been strongly evoked in the negative condition. Although it remains unclear whether *HTR2A* modulates emotion regulation, a recent neuroimaging study indicated that serotonin transporter genotype influences the cognitive reappraisal of negative emotions (Firk et al., 2013). Recent studies have also reported that variations in the serotonin transporter gene-linked polymorphic region are associated with positive emotions (Matsunaga et al., 2010, 2013; De Neve, 2011). Therefore, it is possible that other such serotonin-related gene polymorphisms are associated with the empathic modulation of happiness in both positive and negative situations. Further studies are required to examine this possibility.

It has also been shown that other gene polymorphisms affecting *HTR2A* (rs6313 and T102C) are associated with empathy (Gong et al., 2015). Compared to the T allele, the C allele leads to lower serotonin 2A receptor expression levels and reduces the excitation signal of serotonin in the postsynaptic neuron. Research has further indicated that C allele carriers have a decreased perspective-taking ability and express increased autism-like traits (Gong et al., 2015). Thus, serotonin 2A receptor-associated system deficiencies may also be associated with impaired empathic ability.

### Relationship between susceptibility to others' happiness and subjective happiness

A previous study reported that self-reported susceptibility to others' happiness is negatively associated with subjectively assessed symptoms of depression (Kimura et al., 2007). This suggests that genetic susceptibility to others' happiness (variations in the *HTR2A* gene) is associated with mental health and subjective happiness. However, our questionnaire results indicate that no such association between subjective happiness and *HTR2A* genotype exists. One of the reasons for this null finding may be the presence of gene × environment interactions. A previous study using patients 6 months after lumbar disc surgery reported an *HTR2A* gene × environment interaction (Lebe et al., 2013). Lebe and colleagues found that, in pain-free states, there were no significant differences in self-reported depressive symptoms between A allele carriers and individuals with the GG genotype. However, when the intensity of pain experienced was high, A allele carriers became highly depressive. Because it is believed that *HTR2A* A allele carriers have greater sensitivity to negative stimuli (Lee and Ham, 2008), pain after lumbar surgery may modulate the association between *HTR2A* polymorphisms and depression. Considering this phenomenon, the environment or the positivity of proximal events may influence the association between *HTR2A* polymorphisms and subjective happiness. It has been shown that the repeated experience of positive events elevates our levels of subjective happiness (Seligman et al., 2005; Otake et al., 2006; Schimmack, 2008). Therefore, it is possible that subjective happiness levels of individuals with the *HTR2A* G allele become significantly higher than those of individuals with the AA genotype. However, this may only happen when they experience a series of many positive events. In the present study, we had no data that allowed us to determine whether the participants had experienced many positive events before completing the questionnaire.

### Limitations

Our study has several limitations. First, because only women were included in the fMRI experiment, we cannot generalize the fMRI results to men. For example, the hypothetical situations used in the fMRI study included a marriage proposal scenario. This scenario might not be perceived as realistic because men are more likely than women to propose marriage. However, it is important to note that the questionnaire experiment did not indicate any sex differences in the ability to harbor happiness-related empathy. Nonetheless, replicating the fMRI experiment in both men and women is necessary to draw any strong conclusions. Second, in our questionnaire experiment, we selected one occasion (personal relationship) from 10 occasions used in the fMRI experiment. However, it is unclear whether *HTR2A* polymorphism influences all 10 occasions. It is possible that there are some events that *HTR2A* does not influence. Thus, more detailed analyses may provide us with more information regarding the association between the serotonergic system and empathy. Third, happiness-related empathy may be influenced by interpersonal closeness. For example, a previous neuroimaging study reported that positive feeling-related brain activation in the striatum during empathetic concern was enhanced by an experimental manipulation of target familiarity (Kawamichi et al., 2013b). Although we recruited pairs of actual friends for the present study, it is possible that brain responses differed according to the closeness between the pair of friends, and that there is a difference in happiness-related empathy between romantic partners and strangers. Thus, replication of the study considering such interpersonal closeness will serve to clarify our findings in the future. Fourth, although the present study revealed lateralization of the brain's activity (right temporal pole activity was associated with *HTR2A* polymorphisms), we cannot conclude the *HTR2A* polymorphism-associated brain activity is lateralized due to the small sample size of the fMRI experiment. Because previous neuroimaging studies have reported the effects of specific functions of right brain regions in the mentalizing/theory-of-mind system on empathic abilities (Silani et al., 2013; Hillis, 2014), it is possible that there is a specific connectivity in the mentalizing/theory-of-mind system or specific causal relationships that underlie affective processes. Thus, further studies focusing on this lateralization of function using psycho-physiological interaction analysis or dynamic causal modeling are warranted.

## Conclusions and future directions

The present study shows for the first time that *HTR2A* polymorphisms are associated with the effect of sharing happiness via modulating activity in the mentalizing/theory-of-mind network. Susceptibility to the happiness of others may be associated with psychological and physiological well-being (Kimura et al., 2007). Based on the proposed biological mechanisms underlying susceptibility to others' happiness (the mentalizing/theory-of-mind network and *HTR2A*), future studies may be able to establish effective psychological and physiological interventions to increase sensitivity to the happiness of others in patients with depression, autism spectrum disorders, and various other conditions. Furthermore, this type of intervention exerts additional physical health-promoting effects. The current findings may also contribute to the development of methods for the early and efficient detection of genetic and neural predispositions to depression and autism spectrum disorder, which are known to have sexually dimorphic prevalence and are associated with serotonin neurotransmission. Thus, the present findings may also be applicable to other scientific and medical fields.

## Author contributions

MM, HK, and HO: conceived and designed the fMRI experiments; MM, KI, YO, YN, MO, and HY: conceived and designed the questionnaire experiments; MM, TU, RH, ES, FK, and KS: performed the fMRI experiments; KI, YO, YN, and MO: performed the questionnaire experiments; MM, HK, KI, and YO; analyzed the data; MM, KI, YO, YN, HY, and HO: wrote the paper.

### Conflict of interest statement

The authors declare that the research was conducted in the absence of any commercial or financial relationships that could be construed as a potential conflict of interest. The reviewer KM and handling Editor declared their shared affiliation.
